# Blue rubber bleb nevus syndrome coexisted with intestinal intussusception: a case report

**DOI:** 10.11604/pamj.2014.17.212.3598

**Published:** 2014-03-17

**Authors:** Yuanjie Wang, Xiaojun Zhao, Xiaolan You

**Affiliations:** 1 Department of Gastroenterology Surgery, Taizhou People's hospital, Taizhou, China

**Keywords:** Blue rubber bleb nevus syndrome, gastrointestinal bleeding, jejunojejunal intussusception, intraoperative endoscopy, papule

## Abstract

Blue Rubber Bleb Nevus Syndrome (BRBNS) is an uncommon congenital disorder characterized by sporadic venous malformation which mainly occurs in skin and alimentary canal. Here, we report a BRBNS patient with concomitant intestinal intussusception who diagnosed by intraoperative endoscopy and ultimately managed using surgical resection. A 19-year-old boy was referred to urgent surgery for acute melena and stomachache. He had used to be a long-term iron user for undiagnosed chronic anemia and papules. Abdominal CT on admission demonstrated the presence of intestinal intussusception. The following exploratory laparotomy and intraoperative endoscopy revealed multiple gastrointestinal hemangiomas. The postoperative course was uneventful and pathological examination certified multiple cavernous hemangiomas in the resected gastrointestines.

## Introduction

Blue Rubber Bleb Nevus Syndrome (BRBNS) is an uncommon congenital cutaneous venous malformation coexisting with similar visceral lesions mostly involving gastrointestine and occasionally affecting other internal organs [1, 2]. The main hazardous of BRBNS includes recurrent hemorrhage and secondary anaemia for the ruptured hemangiomas [1]. We presented here a case of BRBNS with concomitant jejunojejunal intussusception explored via intraoperative endoscopy and managed surgically.

## Patient and observation

**Figure 1:**
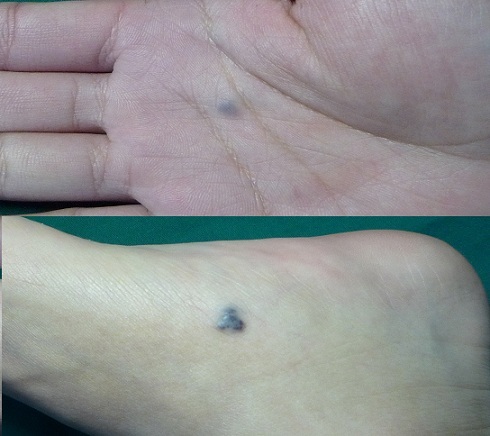
Multiple hemangiomas on extremities

**Figure 2:**
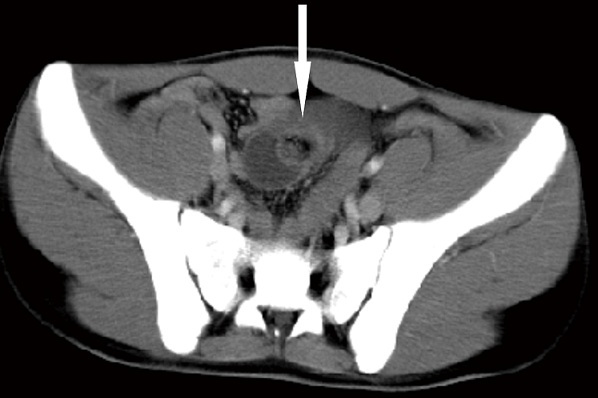
Typical sign of intestinal intussusception in abdominal CT scan: concentric circle (white arrow)

A 19-year-old Chinese boy was referred to our urgent surgery for a week-long melena and abdominal pain. In the past four years, he had persisted iron-supplement orally for unexplained recurrent episodes of hypochromic anemia. Physical examination revealed dark bluish, compressible and refillable, multiple papules with definite boundary on trunk and extremities (Figure 1), as well as fixed tenderness in lower abdomen without guardness. Abdominal computed tomography on admission demonstrated the presence of intestinal intussusception according to the typical sign of concentric circle (Figure 2), thus an exploratory laparotomy was determined.

**Figure 3:**
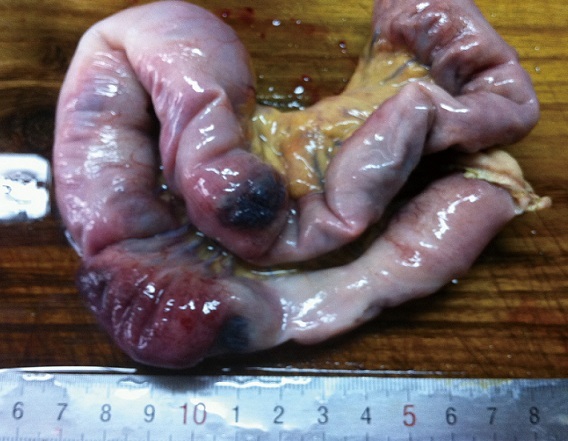
Gastric angeioma discovered via intraoperative gastroduodenoscopy

**Figure 4:**
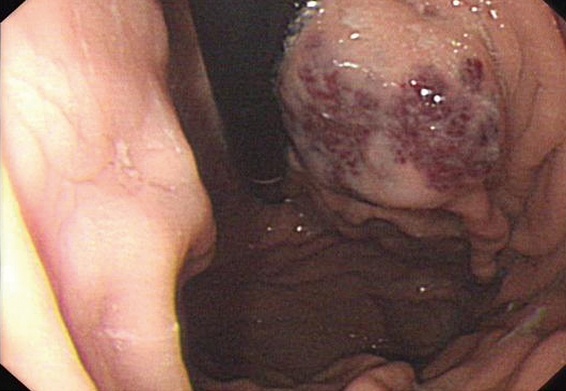
Blue rubber blebs in excised small intestine

**Figure 5:**
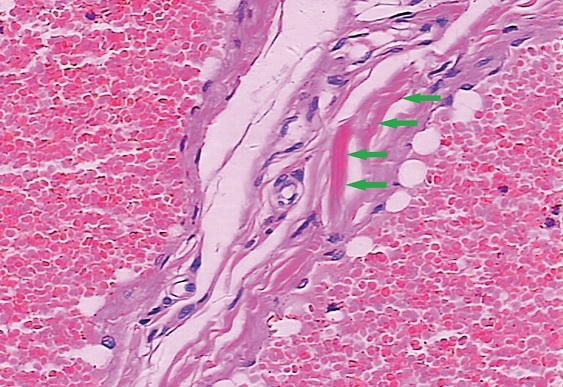
Microscopically, the dilated blood vessel was alternately covered with collagenous fibers (blue arrow) and a single layer of endothelial cells (H&E ×200)

After the upper mid-line incision, a 30-centimeter-long jejunojejunal intussusception without apparent necrosis about 150cm beyond the ligament of Treitz was identified. During the course of tentative manual reduction, two serosal hemangiomas on the incarcerated intestinal was detected (Figure 3), which necessitated the following removal of incarcerated intestine. So far, the diagnosis of BRBNS was defined according to distinct multifocal dermatic and intestinal hemangiomas. To exclude the potential gastrointestinal venous malformation anywhere else, intraoperative esophagogastroduodenoscopy was performed and then a wedge gastrectomy was carried out because of a isolated angioeoplasm found in gastric lumen (Figure 4). Subsequently, an innocent colon was confirmed via intraoperative transanus colonoscopy. Because there was no disinfected enteroscope available during operation, the scheme of intraoperative transparietal enteroscopy was abandoned. Postsurgery, skull and chest CT scanning was utilized and no further lesion was observed. The postoperative course was uneventful and pathological examination revealed multiple cavernous hemangiomas in the resected gastrointestines and eventually the patient recovered uneventfully (Figure 5).

## Discussion

BRBNS is an uncommon congenital venous malformation in skin, gastrointestinal tract and occasionally other internal organs (e.g., nerve and genital system) [1–3]. The distinct pathologic features of BRBNS include the presence of dilated capillaries lined with flat endothelial cells and interrupted tissue matrix, and the resulting formation of cavernous hemangiomas [4, 5]. Because of the blood loss from ruptured hemangiomas in gastrointestinal tract, patients may present with chronic anemia and melena [1]. Managements for these cases always include fundamental symptomatic treatment and selective argon plasma coagulation, band ligation, sclerotherapy or YAG laser [5, 6]. Whereas, in ongoing bleeding condition or the case of intestinal intussusception, the surgical removal of lesions is always obigatory. Thus, a precise and comprehensive positioning diagnosis via pre- or intra- operative endoscopy, particularly transparietal enteroscopy [7], renews significance.

## Conclusion

BRBNS should be a differential diagnosis in patients with unexplained gastroenterology bleeding and papules. Furthermore, intraoperative endoscopy should be utilized to exclude the insidious gastrointestinal BRBNS lesion thoroughly when laparotomy was imperative.
